# Translating words into actions in working memory: The role of spatial-motoric coding

**DOI:** 10.1177/17470218221079848

**Published:** 2022-03-03

**Authors:** Guangzheng Li, Richard J Allen, Graham J Hitch, Alan D Baddeley

**Affiliations:** 1 School of Education Science, Jiangsu Normal University, Xuzhou, China; 2 School of Psychology, University of Leeds, Leeds, UK; 3 Department of Psychology, University of York, York, UK

**Keywords:** Working memory, following instructions, action planning, motor coding, enactment

## Abstract

Research from a working memory perspective on the encoding and temporary maintenance of sequential instructions has established a consistent advantage for enacted over verbal recall. This is thought to reflect action planning for anticipated movements at the response phase. We describe five experiments investigating this, comparing verbal and enacted recall of a series of action–object pairings under different potentially disruptive concurrent task conditions, all requiring repetitive movements. A general advantage for enacted recall was observed across experiments, together with a tendency for concurrent action to impair sequence memory performance. The enacted recall advantage was reduced by concurrent action for both fine and gross concurrent movement with the degree of disruption influenced by both the complexity and the familiarity of the movement. The results are discussed in terms of an output buffer store of limited capacity capable of holding motoric plans for anticipated action.

The concept of working memory as a limited capacity system for maintaining and processing information in the service of complex thought and action is widely held (e.g., Baddeley et al., 2021; Barrouillet & Camos, 2021; Cowan et al., 2021; Logie et al., 2021; Oberauer, 2021; Vandierendonck, 2021). An important aspect of working memory concerns its involvement in the planning and control of behaviour (Baddeley, 2007; Baddeley & Hitch, 1974; Miller et al., 1960). Indeed, one so far underexplored role of working memory may lie in the representation and generation of action (Rosenbaum & Feghhi, 2019).

A good example of this is the practical question of how people turn verbal instructions into actions. This involves the mapping of phonological, syntactic, and semantic processing onto the performance of a sequence of controlled actions, presumably reflecting visual, spatial, tactile, and motor processing. Early research on the following instructions resulted from the study of clinical tests devised to assess possible impairments of syntax in neuropsychological patients. De Renzi and Vignolo (1962), e.g., developed the Token Test as a means of detecting grammatical processing deficits in aphasic patients. The test involves a series of coloured shapes and the requirement to follow instructions increasing in syntactic complexity from simple, e.g., “Touch the red square,” to more complex “Before touching the yellow circle take out the red square.” However, it later became clear that in addition to syntactic comprehension, aspects of short-term memory were also involved in the tasks. Lesser (1976), e.g., showed correlations between Token Test performance and verbal, visual, and motor aspects of short-term memory while patient PV with a dense but specific verbal short-term memory deficit performed very poorly on the Token Test despite subsequent evidence of relatively normal syntactic comprehension (Vallar & Baddeley, 1984).

More recently, considerable attention has been paid to the potential role of working memory in children’s ability to follow instructions in educational activities (Gathercole et al., 2006; also, Engle et al., 1991). This led to a laboratory-based research in which children aged 5–6 years were given analogues of classroom instructions, such as “Touch the green ruler, then pick up the red pencil and put it in the blue box.” (Gathercole et al., 2008). In line with earlier findings in young adults (Koriat et al., 1990), children’s performance was enhanced when they were required to carry out the target activities as compared with simply recalling them verbally. This *enactment advantage* is a robust effect and has since been widely replicated (e.g., Allen & Waterman, 2015; Jaroslawska et al., 2016, 2021; Lui et al., 2018; Makri & Jarrold, 2021; Waterman et al., 2017; Yang et al., 2019, 2021 see Allen et al., 2022, for a review). Conditions in these experiments are typically blocked and it seems that the anticipation of subsequent enaction generates motor representations during encoding and these provide extra support for the performance (Koriat et al., 1990).

Yang et al. (2014, 2016) used dual-task methodology to explore the potential contribution of different components of working memory, as described by the multicomponent model (Baddeley, 1986; Baddeley & Hitch, 1974), to the enacted recall advantage in the following instructions. The addition of concurrent tasks during encoding designed to load on domain-specific verbal (repetition of a three-digit number) or visuospatial working memory (spatial tapping of a pattern on a hidden response board or keypad), or domain-general executive control (backward counting in decrements of three), disrupted memory for instructions but left the enactment advantage intact. It seems therefore that while each of these components of working memory contributes to understanding and remembering instructions, none of them is primarily responsible for the enhanced performance observed when the instructions are enacted rather than verbally recalled. This is consistent with the view that the enactment advantage stems from a separable motoric component of working memory.

A related phenomenon is the observation that physical enactment at encoding can facilitate later memory performance. This has been widely studied in episodic long-term memory paradigms examining recall or recognition for the lists of actions and objects and has been claimed to indicate the activation of spatial-motoric action representations (for reviews, see Engelkamp, 1998; Engelkamp & Zimmer, 1989; Kormi-Nouri, 1995; Logie et al., 2001). A similar benefit of self-enactment during encoding has been observed following a short series of instructions in a working memory context. Allen and Waterman (2015) observed this when the performance was tested by verbal recall but found that the benefit disappeared when the performance was tested by enactment. This interactive effect of enactment at encoding and recall can be readily interpreted in terms of the generation of motor representations in working memory. When the instructions are enacted during their presentation, the motor representations generated will boost verbal recall and enactment. When the instructions are not enacted during presentation, motor representations are not generated with the result that verbal recall does not benefit. In support of this, there is some evidence that encoding-based enactment effects can be reversed by concurrent motor activity (Plancher et al., 2019). Similar findings to those of Allen and Waterman (2015) have been observed in children aged 7–10 years (Jaroslawska et al., 2016; Waterman et al., 2017), though older adults do not seem to benefit from enactment at encoding (Coats et al., 2021; Jaroslawska et al., 2021).

Early evidence concerning the motoric component of working memory came primarily from dual-task studies that showed a double dissociation between short-term memory for configurations of bodily movements, such as clenching the fist and movements towards external spatial locations (Smyth et al., 1988; Smyth & Pendleton, 1989). In these experiments, different types of concurrent movement were performed during the encoding phase of tasks assessing memory span for different types of action. In one case, squeezing and releasing the grip of the hands disrupted memory span for manual configurations but had no effect on span for movements to spatial locations. Conversely, tapping a spatial pattern disrupted span for movements to locations but had no effect on span for configurations of the hand (Smyth & Pendleton, 1989). Based on these and other similar findings, Smyth and Pendleton argued for the existence of a motor store in working memory capable of holding and reproducing configural bodily movement, distinct from the visuospatial sketchpad supporting spatially guided movement. This view would fit with a role for the motor system in working memory for actions (Cortese & Rossi-Arnaud, 2010; Rossi-Arnaud et al., 2004).

The form of motoric representation generated when following verbal instructions were examined in a series of dual-task experiments by Jaroslawska et al. (2018). They studied the effect of performing a repetitive sequence of movements during presentation of the instructions that were subsequently either verbally recalled or physically enacted. The repetitive movements were either “fine,” involving a repeated *palm-fist-point* configuration sequence performed by the hand (taken from Smyth & Pendleton, 1989), or “gross,” involving a sequence of spatially directed forearm movements (see Jaroslawska et al., 2018, Figure 2). Each type of movement impaired recall performance, but gross movements removed the enactment advantage, whereas fine left it intact. Jaroslawska et al. (2018) interpreted these observations as indicating that the motoric component of working memory is dedicated to the temporary maintenance of gross but not fine motoric representations of planned action sequences.

This study used a dual-task approach to investigate in more detail the form of memory storage system on which the enactment effect depends. We began by attempting to replicate the distinction between fine and gross motor movements reported by Jaroslawska et al. Their conclusion was based on a post hoc comparison between separate experiments, and we aimed to improve on this by comparing the disruptive effects of fine and gross movements directly in the same experiment. Furthermore, a potential problem in interpreting both Jaroslawska et al.’s study and those of Smyth and Pendleton lies in interpreting the gross–fine distinction. This might suggest a single dimension of precision. However, there are a number of ways in which a sequence of unrelated hand gestures may differ from a continuous pattern of arm movements that go beyond the different potential of the hand and arm for precise action. These include the role of spatial location, degree of continuity, the complexity and familiarity of the actions, the potential social significance of hand movements, and the nature and range of possible configurations of the hand and the arm. Rather than try to separate these, we opted for a relatively simple motor distinction, that of tracing a spatial path on a gross scale using an arm versus tracing the same path on a fine scale using a finger, leaving for future investigation the other dimensions on which the concurrent tasks used by Smyth and Pendleton (1989) and Jaroslawska et al. (2018) may have differed.

The remaining four experiments in this series then moved on to explore further dimensions of movement type, namely complexity and familiarity. We regard concurrent actions as serving a system-specific disruptive role that is broadly analogous to that of articulatory suppression in the phonological loop. In that case, the repeated utterance of a single simple word such as “the” is assumed to impair concurrent articulatory and phonological processing while placing only a minimal load on executive resources. The system can then be explored further by systematically manipulating the concurrent task, e.g., by increasing its complexity (see e.g., Baddeley & Hitch, 1974) or content (Mate et al., 2012). In the present investigation, our exploration varied both the familiarity and complexity of concurrent movements with the aim of beginning to map out the characteristics of the hypothetical system assumed to underpin the role of enactment in working memory.

We report five experiments exploring whether memory for instructions, and particularly when these require enactment at recall, is influenced by manipulations along different dimensions of movement. We started with a simple comparison of finger- (fine) and arm-based (gross) movement (Experiment 1), before moving on to examine the effects of concurrent movement complexity, either with the finger (Experiment 2) or arm (Experiment 3), and finally familiarity, again implemented either with the finger (Experiment 4) or arm (Experiment 5).

## Experiment 1

We began by exploring whether concurrent performance of fine versus gross motor tasks would differentially impact on memory for action–object instruction sequences in general, and on any observed enacted recall advantage in particular. Using a variant of the Gathercole et al. (2008) following instructions task, Jaroslawska et al. (2018, Experiments 2–3) found that concurrent gross motor movement abolished the difference in accuracy between verbal and enacted recall. Such a pattern was not observed in Jaroslawska et al. (2018, Experiment 1) when using an entirely different, fine motor movement task.

Our first experiment aimed to replicate and extend this finding. Rather than using very different movement patterns in the fine and gross conditions, we equated their form and complexity, with participants required to draw a “W” pattern in the air using either their finger (fine) or arm (gross movement). Thus, it can reasonably be assumed that any difference in the performance between these conditions reflects this fine–gross movement distinction rather than other forms of potentially confounding variation (e.g., complexity of action or sequence).

In this and all subsequent experiments, we examined the impacts of the concurrent movement tasks on a version of the following instructions paradigm in which a set of actions are arbitrarily paired with geometric objects on each trial (Allen et al., 2020; Allen & Waterman, 2015; Waterman et al., 2017). This method has the advantage of using objects with minimal prior affordance or associated movement patterns, thus emphasising the requirement to encode new action–object associations within working memory. It also equates the number of actions and objects in the experimental pool and uses an increased number of distinct actions while avoiding repetition of features within any one trial (cf. Jaroslawska et al., 2018). For the sake of simplicity, we use a set length of four object pairs per sequence, following the method implemented by Jaroslawska et al. (2018) and Cowan’s (2001) identification of a working memory capacity limit of approximately four chunks of information.

### Method

#### Participants

In total, 30 right-handed adults (aged 20–25 years, *M* = 22.73 years, *SD* = 1.86; 25 females and 5 males) took part in this experiment. All were Chinese native speakers at the Jiangsu Normal University. All participants had normal or corrected-to-normal vision and hearing, and no evidence of current or past major neurological disorders or psychiatric disorder. No participants were previously involved in any similar experiment. Based on the enacted recall advantage (*d* = 1.14) observed in the baseline condition in Allen and Waterman (2015), we anticipated a large effect size (*d* ⩾ 0.8) in the present experimental series. G*power (Faul et al., 2009) indicated a required sample size of *N* = 23 to detect an effect size of *d* = 0.8 at α < .05 with 95% power.

The study was approved by the Ethics Committees of Jiangsu Normal University and Department of Psychology, University of York. Informed consent was obtained from all participants prior to testing. These ethical and informed consent requirements were also met for the subsequent reported experiments.

#### Materials

Six shapes (circle, cross, square, star, sun, and triangle) each depicted as a black solid against a rectangular white background measuring 5 × 5 cm^2^ were pasted onto cork coasters double-sided to make them easy to manipulate. They were pseudorandomly arranged on a desktop in front of the participant. The arrangement was different for each participant and remained constant throughout the experiment. Six actions (drag, flip, lift, push, spin, and touch) were combined with the shapes to form a pool of 36 action–object pairs. Each trial consisted of the spoken presentation of four actions and objects selected randomly without replacement from the pool (e.g., *flip the cross, drag the triangle, push the square, lift the star*). Six blocks of such trials were generated, 1 for each experimental condition, with 2 practice, and 10 test trials in each block.

#### Design and procedure

A 2 (Recall mode: verbal, enacted) × 3 (Concurrent task: no task, finger movement, and arm movement) repeated measures design was used. Each of the six conditions was performed in a separate block of trials. The order of blocks was counterbalanced across participants, with concurrent task conditions nested within recall mode. The dependent variable was the mean proportion of action–object pairs recalled in the correct serial position per trial.

At the beginning of the experiment, subjects were familiarised with the shapes and their verbal labels, and with each physical action. Following this, they were given practice on the secondary tasks. To control for the amplitude of the concurrent action, participants were required to place their right index fingertip (finger movement condition) or right arm (arm movement condition) at eye level and draw a palm-sized “W” from left to right, with its base at the level of chin. In the former, the index finger was positioned immediately to the right of the eye, and in the latter, the arm was extended frontally. The speed of the movement was self-determined and hence varied somewhat between individuals. However, Jaroslawska et al. (2018) found that this was of little significance. After finishing a movement, participants were required to return their finger or arm to the original position and continue the concurrent task until they were asked to recall the sequence of instruction. On each trial, the performance of the concurrent task movement began 5 s before sequence presentation. Each sequence of instructions was auditorily presented from a notebook computer, at a rate of approximately 3 s for each action–object pair, followed by a 3-s pause. After completing each instruction sequence, a reminder (“Recall Now”) was presented, 1 s after the presentation of the last instruction. Participants were told not to repeat the instructions aloud, touch, operate, or move the objects during encoding. They were required to listen to the four action–object phrases while doing nothing (no concurrent task), while using their right index fingertip (finger movement condition), or their right arm (arm movement condition) to draw the letter “W” in the air. In the recall stage, participants either verbally repeated the instructions (verbal recall) or physically performed the actions (enacted recall). A video camera was set up behind the participants to record the entire experiment. At the end of each trial, the shapes were restored to their original positions.

### Results

Following the previous work (e.g., Allen & Waterman, 2015; Gathercole et al., 2008; Jaroslawska et al., 2018), the performance was indexed by the mean proportion of action–object pairs recalled in the correct serial position in this and all subsequent experiments. A summary of outcomes from the analyses scoring actions and objects as separate features is provided in the online Supplementary Materials. The data are publicly available on the Open Science Framework [https://osf.io/gdtwh/]. All analyses were carried out in JASP 0.14.1 (JASP Team, 2021). We report the results of both frequentist and Bayes Factor (BF) analytic approaches. BF analysis computes the strength of evidence for the presence (or absence) of an effect and can therefore be used to assess equivalence between conditions. In this study, we report the *BF*_10_ for each main effect and interaction. A *BF*_10_ value above 1 indicates evidence of an effect, whereas a *BF*_10_ value below 1 (or alternatively, a *BF*_01_ value, calculated as 1/*BF*_10_, that is larger than 1) indicates evidence of no effect. However, it is generally viewed that any *BF*_10_ or *BF*_01_ between 1 and 3 only provides anecdotal evidence (Jeffreys, 1961; Schönbrodt & Wagenmakers, 2018), and we adopt this classification here.

First, the full experimental design was analysed using a 3 × 2 (concurrent task × recall mode) repeated measures analysis of variance (ANOVA). This was then followed with two 2 × 2 repeated-measures ANOVA, comparing the no-task condition with each of the concurrent task conditions, to establish whether any enacted recall advantage was affected by each task in turn. Finally, following Jaroslawska et al. (2018), paired samples *t*-tests were carried out, examining the difference between enacted and verbal recall conditions in each concurrent task condition.

**Figure 1.**
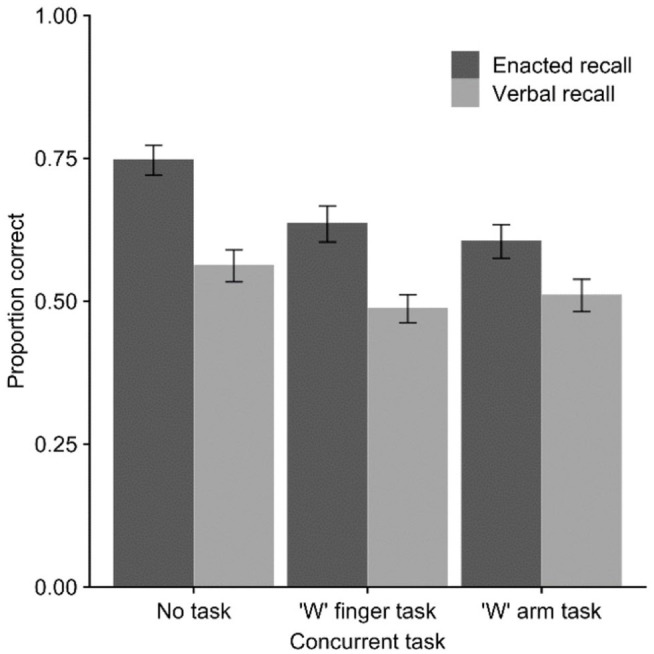
Mean proportion of action–object pairs correct (with *SE*) in Experiment 1 across verbal and enacted recall modes and concurrent movement task conditions.

Figure 1 shows the performance for each recall mode in the three concurrent task conditions. The overall 3 × 2 ANOVA indicated a significant effect of recall mode, *F*(1, 29) = 44.64, *MSE* = 0.91, *p* < .001, $\eta_{p}^{2}=.61$ , *BF*_10_ > 10,000, with superior performance under enacted (*M* = 0.66, *SE* = 0.02) relative to verbal (*M* = 0.52, *SE* = 0.02) recall conditions. The main effect of concurrent task was significant, *F*(2, 58) = 23.74, *MSE* = 0.18, *p* < .001, $\eta_{p}^{2}=.45$ , *BF*_10_ > 10,000, with further comparisons revealing that recall in the no-task condition (*M* = 0.65, *SE* = 0.02) was higher than in both the finger (*M* = 0.56, *SE* = 0.02), *t*(29) = 9.15, *p* < .001, *d* = 1.67, *BF*_10_ > 10,000, and arm (*M* = 0.56, *SE* = 0.02), *t*(29) = 5.32, *p* < .001, *d* = 0.97, *BF*_10_ = 3,239, movement conditions, which did not themselves differ, *t*(29) = 0.21, *p* = .84, *d* = 0.04, *BF*_10_ = .144. The interaction between recall mode and concurrent task was also significant, *F*(2, 58) = 3.23, *MSE* = 0.031, *p* = .047, $\eta_{p}^{2}=.10$ , *BF*_10_ = 0.88, reflecting a small reduction in the action advantage in the dual-task conditions, though this was not supported by the Bayesian analysis which slightly favoured the null (*BF*_01_ = 1.14).

For the 2 × 2 ANOVA comparing no-task condition with finger movement, there was an effect of recall mode, *F*(1, 29) = 56.07, *p* < .001, $\eta_{p}^{2}=.66$ , *BF*_10_ > 10,000, and concurrent task, *F*(1, 29) = 83.70, *p* < .001, $\eta_{p}^{2}=.74$ , *BF*_10_ > 10,000, but no interaction, *F*(1, 29) = 2.00, *p* = .17, $\eta_{p}^{2}=.07$ , *BF*_10_ = 0.92. For the comparison of no task with arm movement, there was an effect of recall mode, *F*(1, 29) = 30.93, *p* < .001, $\eta_{p}^{2}=.52$ , *BF*_10_ > 10,000, concurrent task, *F*(1, 29) = 28.29, *p* < .001, $\eta_{p}^{2}=.49$ , *BF*_10_ > 10,000, and a significant interaction, *F*(1, 29) = 5.69, *p* = .024, $\eta_{p}^{2}=.16$ , *BF*_10_ = 1.71, though this was not strongly supported by the BF.

Finally, comparison of recall modes indicated an advantage for enacted versus verbal recall in the no-task condition, *M* = 0.75 versus *M* = 0.56, *t*(29) = 7.25, *p* < .001, *d* = 1.32, *BF*_10_ > 10,000, in the finger task condition, *M* = 0.64 versus *M* = 0.49, *t*(29) = 5.71, *p* < .001, *d* = 1.04, *BF*_10_ = 5,466, and in the arm task condition, *M* = 0.60 versus *M* = 0.51, *t*(29) = 2.58, *p* = .015, *d* = 0.47, *BF*_10_ = 3.16.

### Discussion

This first experiment replicated the advantage for enacted over verbal recall found previously in memory for instruction sequences (e.g., Allen & Waterman, 2015; Gathercole et al., 2008; Jaroslawska et al., 2016; Yang et al., 2016). The main effect of concurrent task was significant overall. This might be taken to indicate a general dual-task effect across all conditions, possibly reflective of executive control. Alternatively, a degree of spatial-motor coding may be involved in encoding and maintaining sequences of instructions in working memory, regardless of response mode.

There was also a marginal response type × concurrent task interaction in the overall ANOVA, suggesting a component specific to preparing an enacted response that might be broadly spatial-motoric in nature. Separate comparison of each concurrent task with the no-task condition broadly replicated the findings of Jaroslawska et al. (2018), who compared “fine” and “gross” tasks in separate experiments and analyses. Thus, there was no recall × task interaction when examining finger movement, but we did observe such an interaction when examining arm movement. However, it should be noted that the BF support was weak in each case, with *BF*_10_ or *BF*_01_ always less than 3. The enacted recall advantage also remained intact in all three concurrent task conditions (unlike the gross movement conditions in Jaroslawska et al., 2018), though it was reduced in the arm movement condition relative to no task or finger movement.

This continued presence of an enacted recall effect in all conditions, and the absence of stronger support for a recall × task interaction would indicate that movement scale is not the only factor that should be considered when exploring how spatial-motor plans are constructed and maintained in working memory. Thus, Jaroslawska et al. (2018) may have overinterpreted their results, which might in fact have reflected other dimensions of the concurrent motor task that covaried with their difference in scale. The following experimental series therefore explored complexity and familiarity as novel dimensions of motor movement that might be important in this context, either as concurrent finger movement (Experiments 2 and 4) or arm movement (Experiments 3 and 5).

## Experiment 2: simple and complex finger movement

Experiment 2 manipulated complexity of concurrent finger movement. Using the analogy of articulatory suppression (Baddeley et al., 2001), it seems likely that increasing the complexity of concurrent movement might increase its disruptive capacity. This could of course reflect a greater load on the central executive component of working memory, in which case we would expect to see a similar impact on both spoken and enacted recall. However, if complex movements place more demands on a separable motor component of working memory, we might expect to see more impact on enacted than spoken recall. We chose as our concurrent task tracing a familiar Chinese character, manipulating complexity by the number of strokes required to write it. The simple motor task involved repeatedly drawing the Chinese character for the number 10, which involves two distinct movements. The complex task used the Chinese character for the number 6, which involves four distinct movements. These characters have equivalent meaning and familiarity to a Chinese population sample.

### Method

#### Participants

There were 24 right-handed adults (aged 19–27 years, *M* = 22.42 years, *SD* = 1.89; 22 females and 2 males). All were Chinese native speakers at the Jiangsu Normal University, with normal or corrected-to-normal vision and hearing, and no evidence of current or past major neurological disorders or psychiatric disorder. No participants were previously involved in any similar experiment.

#### Materials

The materials from Experiment 1 were used again here.

#### Design and procedure

Each experiment used a 2 × 3 repeated measures design combining recall mode (verbal or enaction) with concurrent task condition (no-task baseline, simple movement [tracing Chinese character “十”], and complex movement [tracing Chinese character “六”]). Participants were required to trace the characters in the air using the right index finger (fine movement). Each of the six conditions was performed in a separate block of trials. The order of blocks was counterbalanced across participants, with concurrent task conditions nested within recall mode. The primary dependent variable was the mean proportion of action–object pairs recalled in the correct serial position per trial.

### Results

**Figure 2.**
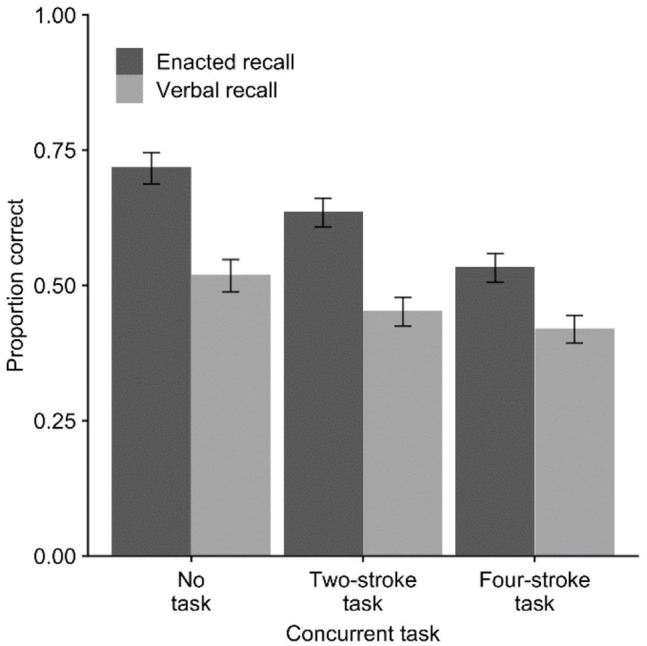
Mean proportion of action–object pairs correct (with *SE*) in Experiment 2 across verbal and enacted recall modes and concurrent movement task conditions.

Figure 2 shows the performance for each recall mode in the three concurrent task conditions. The overall 3 × 2 ANOVA indicated a significant effect of recall mode, *F*(1, 23) = 50.67, *p* < .001, $\eta_{p}^{2}=.69$ , *BF*_10_ > 10,000, with superior performance under enacted (*M* = 0.63, *SE* = 0.02) relative to verbal (*M* = 0.46, *SE* = 0.03) recall conditions. The main effect of concurrent task was significant, *F*(2, 56) = 61.53, *p* < .001, $\eta_{p}^{2}=.73$ , *BF*_10_ > 10,000, with further comparisons revealing that recall in the no-task condition (*M* = 0.62, *SE* = 0.02) was higher than in both the simple, (*M* = 0.54, *SE* = 0.02), *t*(23) = 5.83, *p* < .001, *d* = 1.19, *BF*_10_ > 10,000, and complex, (*M* = 0.48, *SE* = 0.02), *t*(23) = 11.09, *p* < .001, *d* = 2.26, *BF*_10_ > 10,000, movement conditions, which themselves also differed, *t*(23) = 5.26, *p* < .001, *d* = 1.07, *BF*_10_ = 207.11. The interaction between recall mode and concurrent task was also significant, after Greenhouse–Geisser correction, *F*(1.38, 31.50) = 7.17, *p* = .007, $\eta_{p}^{2}=.24$ , *BF*_10_ = 1.80, indicating somewhat greater disruption in the enacted condition, though with relatively weak BF support.

For the 2 × 2 ANOVA comparing no task with two-stroke movement, there was an effect of recall mode, *F*(1, 23) = 55.52, *p* < .001, $\eta_{p}^{2}=.71$ , *BF*_10_ > 10,000, and concurrent task, *F*(1, 23) = 36.23, *p* < .001, $\eta_{p}^{2}=.61$ , *BF*_10_ = 433.14, but no interaction, *F*(1, 23) = 0.81, *p* = .38, $\eta_{p}^{2}=.03$ , *BF*_10_ = .27. For the comparison of no task with four-stroke movement, there was an effect of recall mode, *F*(1, 23) = 42.87, *p* < .001, $\eta_{p}^{2}=.52$ , *BF*_10_ > 10,000, and concurrent task, *F*(1, 23) = 88.70, *p* < .001, $\eta_{p}^{2}=.79$ , *BF*_10_ > 10,000, and a significant interaction, *F*(1, 23) = 7.62, *p* = .011, $\eta_{p}^{2}=.25$ , *BF*_10_ = 2.5.

Finally, comparison of recall modes indicated an advantage for enacted versus verbal recall for all three conditions, in the no-task condition, *M* = 0.72 versus *M* = 0.52, *t*(23) = 6.67, *p* < .001, *d* = 1.36, *BF*_10_ *>* 10,000, in the finger task condition, *M* = 0.63 versus *M* = 0.45, *t*(23) = 7.64, *p* < .001, *d* = 1.59, *BF*_10_ *>* 10,000, and in the arm task condition, *M* = 0.53 versus *M* = 0.42, *t*(23) = 4.21, *p* < .001, *d* = 0.86, *BF*_10_ = 92.64.

### Discussion

Experiment 2 replicated the enacted recall advantage and the overall disruptive effect of concurrent movement observed in Experiment 1. In addition, there was some evidence for an interaction between response mode and concurrent task, with movements during encoding serving to reduce the advantage of enacted over verbal recall. This indicates evidence for a motoric component in working memory that is more critical to encoding for enacted recall. The results also include novel findings regarding motor complexity. Thus, increasing the complexity of a concurrent motor task (from two to four strokes per movement) resulted in greater interference effects in working memory for instruction sequences. This effect was greater for enacted than verbal recall, with a significant recall mode × concurrent task interaction emerging, and a reduced (but still large) enacted recall advantage observed. Taken together these findings suggest that the system responsible for generating spatial-motor movements does contribute to working memory, and to the enacted recall advantage in particular, though the continuing emergence of the enactment advantage in all conditions indicates that our manipulation of motor complexity was not sufficient to completely prevent action planning.

## Experiment 3

Experiment 2 established that concurrent finger movement during encoding reduces sequence recall performance in general and impacts particularly on enacted recall, with some indication that this varies with movement complexity. In Experiment 3, we moved to explore the extent to which these findings replicate using a different scale of movement, namely arm movements.

### Method

#### Participants

Overall, 24 right-handed adults (aged 20–25 years, *M* = 22.88 years, *SD* = 1.72; 16 females and 8 males) took part in Experiment 3.

#### Materials, design, and procedure

This experiment used the same methodology as Experiment 2. The only difference was that the concurrent movements were performed by the arm.

### Results

**Figure 3.**
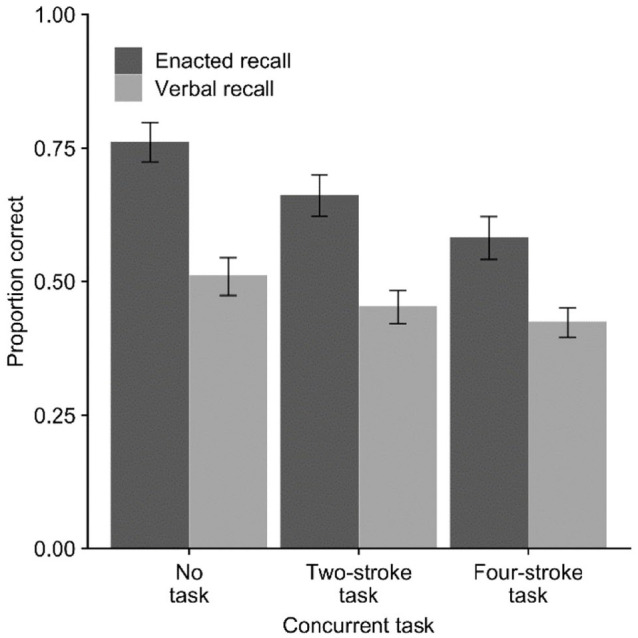
Mean proportion of action–object pairs correct (with *SE*) in Experiment 3 across verbal and enacted recall modes and concurrent movement task conditions.

Mean proportion of action–object pairs recalled in the correct serial position is displayed in Figure 3. The overall 3 × 2 ANOVA indicated a significant effect of recall mode, *F*(1, 23) = 41.50, *p* < .001, $\eta_{p}^{2}=.64$ , *BF*_10_ > 10,000, with superior performance under enacted (*M* = 0.67, *SE* = 0.03) relative to verbal (*M* = 0.46, *SE* = 0.03) recall conditions. The main effect of concurrent task was significant, *F*(2, 56) = 57.30, *p* < .001, $\eta_{p}^{2}=.71$ , *BF*_10_ *>* 10,000, with further comparisons revealing that recall in the no-task condition (*M* = 0.64, *SE* = 0.03) was higher than in both the simple, (*M* = 0.56, *SE* = 0.03), *t*(23) = 6.30, *p* < .001, *d* = 1.29, *BF*_10_ > 10,000, and complex, (*M* = 0.50, *SE* = 0.03), *t*(23) = 10.65, *p* < .001, *d* = 2.17, *BF*_10_ > 10,000, movement conditions, which themselves also differed, *t*(23) = 4.34, *p* < .001, *d* = 0.87, *BF*_10_ = 122. The interaction between recall mode and concurrent task was also significant, *F*(2, 56) = 6.86, *p* = .002, $\eta_{p}^{2}=.23$ , *BF*_10_ = 0.78, though this was again not supported by the Bayesian analysis (*BF*_01_ = 1.28).

For the 2 × 2 ANOVA comparing no task with two-stroke movement, there was an effect of recall mode, *F*(1, 23) = 51.15, *p* < .001, $\eta_{p}^{2}=.69$ , *BF*_10_ > 10,000, and concurrent task, *F*(1, 23) = 52.91, *p* < .001, $\eta_{p}^{2}=.70$ , *BF*_10_ = 57.62, but no interaction, *F*(1, 23) = 2.38, *p* = .14, $\eta_{p}^{2}=.09$ , *BF*_10_ = 0.44. For the comparison of no task with four-stroke movement, there was an effect of recall mode, *F*(1, 23) = 37.14, *p* < .001, $\eta_{p}^{2}=.62$ , *BF*_10_ > 10,000, and concurrent task, *F*(1, 23) = 105.33, *p* < .001, $\eta_{p}^{2}=.82$ , *BF*_10_ > 10,000, and a significant interaction, *F*(1, 23) = 14.19, *p* = .001, $\eta_{p}^{2}=.38$ , *BF*_10_ = 1.62. In the latter case, the *F* value and effect size for the interaction were large though it was not strongly supported by the BF.

Finally, comparison of recall modes indicated an advantage for enacted versus verbal recall advantage in the no-task condition, *M* = 0.76 versus *M* = 0.51, *t*(23) = 6.93, *p* < .001, *d* = 1.42, *BF*_10_ > 10,000, in the simple task condition, *M* = 0.66 versus *M* = 0.45, *t*(23) = 6.19, *p* < .001, *d* = 1.26, *BF*_10_ = 7451, and in the complex task condition, *M* = 0.58 versus *M* = 0.42, *t*(23) = 4.48, *p* < .001, *d* = 0.92, *BF*_10_ = 170.

### Discussion

Moving from concurrent finger to arm movement, Experiment 3 closely replicated the outcomes of Experiment 2. We found an enacted recall advantage, a general concurrent movement effect, and an impact of movement complexity. Furthermore, there was an increased motor interference effect for enacted recall relative to verbal recall. The enacted recall advantage remained sizable across conditions, though it somewhat reduced in size when participants performed a more complex concurrent task during encoding.

In both Experiments 2 and 3, while the frequentist analysis produced significant interactions between recall mode and concurrent task in each case, the associated Bayesian analysis only indicated relatively weak positive evidence in the comparison of no task with the more complex task condition. We combined the datasets from Experiments 2 and 3 to derive a larger sample size while also enabling direct comparison of movement scale (finger vs arm movement) as an additional between-subjects factor.

## Combined analysis of Experiments 2 and 3

A 2 × 3 × 2 (recall mode × concurrent task × experiment) mixed ANOVA was performed. We observed a significant effect of recall mode, *F*(1, 46) = 88.27, *p* < .001, $\eta_{p}^{2}=.66$ , *BF*_10_ > 10,000, reflecting superior performance under enacted (*M* = 0.65, *SE* = 0.02) compared to verbal (*M* = 0.46, *SE* = 0.02) recall. The main effect of concurrent task was also significant, *F*(2, 92) = 118.65, *p* < .001, $\eta_{p}^{2}=.72$ , *BF*_10_ > 10,000, with further comparisons showing recall in the no-task condition (*M* = 0.63, *SE* = 0.02) to be higher than in both the simple movement, (*M* = 0.55, *SE* = 0.02), *t*(47) = 9.41, *p* < .001, *d* = 1.40, and complex movement, (*M* = 0.49, *SE* = 0.02), *t*(47) = 13.95, *p* < .001, *d* = 2.01, conditions, which also differed, *t*(47) = 7.12, *p* < .001, *d* = 1.03 (all *BF*_10_ > 10,000). The interaction between recall mode and concurrent task was also significant, *F*(2, 92) = 13.69, *p* < .001, $\eta_{p}^{2}=.23$ , *BF*_10_ = 9.03, with enacted recall being more disrupted by concurrent movement than verbal recall. However, there was no main effect of movement amplitude (finger vs arm) nor did this interact with any other factor (*F* < 1.5, *p* > .2, $\eta_{p}^{2}<.03$ , *BF*_10_ < 1).

For the 2 × 2 × 2 ANOVA comparing no task with two-stroke movement, there was an effect of recall mode, *F*(1, 46) = 104.83, *p* < .001, $\eta_{p}^{2}=.70$ , *BF*_10_ > 10,000, and concurrent task, *F*(1, 46) = 86.84, *p* < .001, $\eta_{p}^{2}=.65$ , *BF*_10_ = 57.62, but no interaction, *F*(1, 46) = 3.18, *p* = .08, $\eta_{p}^{2}=.07$ , *BF*_10_ = 0.36. For the comparison of no task with four-stroke movement, there was an effect of recall mode, *F*(1, 46) = 76.74, *p* < .001, $\eta_{p}^{2}=.63$ , *BF*_10_ > 10,000, and concurrent task, *F*(1, 46) = 191.35, *p* < .001, $\eta_{p}^{2}=.81$ , *BF*_10_ > 10,000, and a significant interaction, *F*(1, 46) = 20.30, *p* < .001, $\eta_{p}^{2}=.31$ , *BF*_10_ = 13.71. Thus, the combined analysis of Experiments 2 and 3 provides no evidence for a recall by concurrent task interaction when examining the simple two-stroke task, but strong evidence for this interaction when using the more complex four-stroke task. However, in neither 2 × 2 analysis was there any main effect of movement amplitude (i.e., finger vs arm) or interaction with any other factor (*F* < 1.5, *p* > .2, $\eta_{p}^{2}<.03$ , *BF*_10_ < 1).

Finally, comparison of recall modes indicated an advantage for enacted versus verbal recall in the no-task condition, *M* = 0.74 versus *M* = 0.51, *t*(47) = 9.56, *p* < .001, *d* = 1.38, the simple movement condition, *M* = 0.65 versus *M* = 0.45, *t*(47) = 9.55, *p* < .001, *d* = 1.38, and the complex movement condition, *M* = 0.56 versus *M* = 0.42, *t*(47) = 6.12, *p* < .001, *d* = 0.88, with *BF*_10_ > 10,000 in all cases.

## Experiment 4: familiar and unfamiliar finger movement

Experiment 4 examined whether a different type of movement dimension, namely familiarity, mirrors the patterns seen with complexity and serves to disrupt performance overall, and the enacted recall advantage. It has been demonstrated that well-learnt, meaningful actions are imitated and performed faster and more accurately relative to novel actions (Hulstijn & van Galen, 1988; Rumiati et al., 2005; Rumiati & Tessari, 2002). In their exploration of handwriting, e.g., Hulstijn and van Galen suggested that units of motor programming vary depending on the nature of the task and the amount of practice and familiarity associated with the movement. Movements consisting of letters can be coded as such, whereas unfamiliar patterns, or familiar patterns with spaces introduced, may be programmed as sequences of individual strokes. Thus, familiar movement sequences might be chunked into larger units, relative to unfamiliar movements (e.g., De Kleine & Van der Lubbe, 2011). Manipulating prior familiarity of concurrent movement therefore offers an alternative way of varying motor load while holding movement pattern complexity constant.

We used either “A” (highly familiar to our participants) or an unfamiliar inverted orientation (“∀”) letter tracing patterns that were otherwise matched in complexity. These were again implemented using finger movements. If making unfamiliar movements places more load on spatial-motoric resources for action planning in working memory, we would expect to see recall mode × concurrent task interactions whereby such movements reduce or remove the enacted recall advantage.

### Method

#### Participants

We tested 24 right-handed adults (aged 19–23 years, *M* = 20.75 years, *SD* = 0.68; 22 females and 2 males). All were Chinese native speakers at the Jiangsu Normal University and had English as their second language. English is also the test subject during the college entrance examination. Thus, each participant is familiar with the letter A. No participants were previously involved in any of the previous experiments.

#### Materials, design, and procedure

This experiment used a 2 (recall mode: verbal, action) × 3 (concurrent task: no task, familiar task; tracing the letter “A”), unfamiliar task (tracing an inverted A, i.e., “∀”), repeated measures design. The same methods as in Experiment 1a were implemented here, with the exception that participants were asked to trace in the air either the letter “A” (familiar movement pattern) or an inverted “A” (unfamiliar movement) during encoding.

### Results

**Figure 4.**
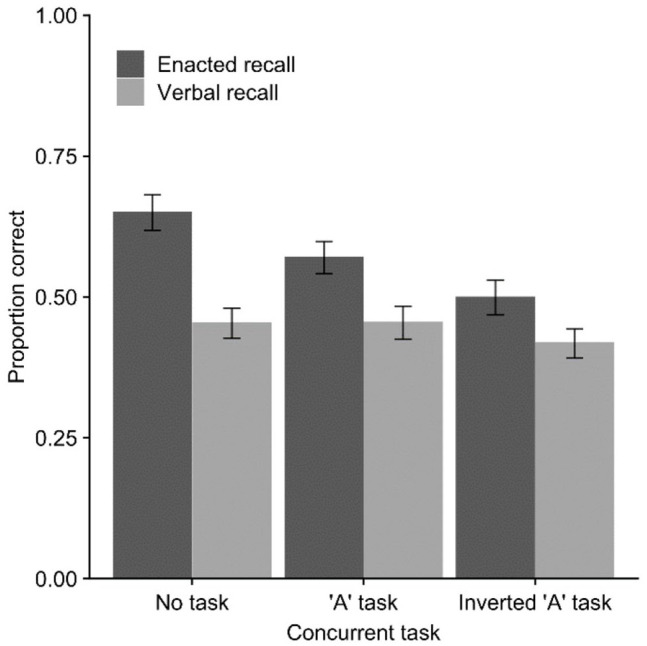
Mean proportion of action–object pairs correct (with *SE*) in Experiment 4 across verbal and enacted recall modes and concurrent movement task conditions.

Figure 4 shows the performance for each recall mode in the three concurrent task conditions. The overall 3 × 2 ANOVA indicated a significant effect of recall mode, *F*(1, 23) = 66.90, *p* < .001, $\eta_{p}^{2}=.74$ , *BF*_10_ > 10,000, with superior performance under enacted (*M* = 0.57, *SE* = 0.03) relative to verbal (*M* = 0.44, *SE* = 0.03) recall conditions. The main effect of concurrent task was significant, *F*(2, 56) = 29.18, *p* < .001, $\eta_{p}^{2}=.56$ , *BF*_10_ > 10,000, with further comparisons revealing that recall in the no-task condition (*M* = 0.55, *SE* = 0.03) was higher than in both the familiar, (*M* = 0.51, *SE* = 0.03), *t*(23) = 3.23, *p* = .002, *d* = 0.66, *BF*_10_ = 7.68, and unfamiliar, (*M* = 0.46, *SE* = 0.03), *t*(23) = 7.61, *p* < .001, *d* = 1.55, *BF*_10_ > 10,000, movement conditions, which themselves also differed, *t*(23) = 4.38, *p* < .001, *d* = 0.89, *BF*_10_ = 2,337. The interaction between recall mode and concurrent task was also significant, *F*(2, 56) = 14.20, *p* < .001, $\eta_{p}^{2}=.38$ , *BF*_10_ = 126.

For the 2 × 2 ANOVA comparing no task with familiar (“A”) movement, there was an effect of recall mode, *F*(1, 23) = 86.25, *p* < .001, $\eta_{p}^{2}=.79$ , *BF*_10_ > 10,000, concurrent task, *F*(1, 23) = 9.72, *p* = .005, $\eta_{p}^{2}=.30$ , *BF*_10_ = 4.70, and the interaction, *F*(1, 23) = 13.12, *p* = .001, $\eta_{p}^{2}=.36$ , *BF*_10_ = 8.45. For the comparison of no task with unfamiliar (inverted “A” movement), there was an effect of recall mode, *F*(1, 23) = 55.61, *p* < .001, $\eta_{p}^{2}=.71$ , *BF*_10_ > 10,000, and concurrent task, *F*(1, 23) = 50.18, *p* < .001, $\eta_{p}^{2}=.69$ , *BF*_10_ > 10,000, and a significant interaction, *F*(1, 23) = 23.73, *p* < .001, $\eta_{p}^{2}=.51$ , *BF*_10_ = 84.42.

Finally, comparison of recall modes indicated an advantage for enacted versus verbal recall advantage in all three conditions, for the no-task condition, *M* = 0.65 versus *M* = 0.45, *t*(23) = 8.58, *p* < .001, *d* = 1.75, *BF*_10_ > 10,000, in the familiar task condition, *M* = 0.57 versus *M* = 0.45, *t*(23) = 6.77, *p* < .001, *d* = 1.38, *BF*_10_ > 10,000, and in the unfamiliar task condition, *M* = 0.50 versus *M* = 0.42, *t*(23) = 3.83, *p* < .001, *d* = 0.78, *BF*_10_ = 39.72.

### Discussion

This experiment replicated the enacted recall advantage, and the effect of concurrent finger movement task found in the experimental series so far. There was also a novel main effect of concurrent movement familiarity, with recall worse when a less familiar (inverted “A”) movement was performed during encoding. We also found response × task interactions both for the familiar and unfamiliar tasks when comparing against the no-task condition, with somewhat stronger evidence in the latter case. Enacted recall effects were apparent in all concurrent task conditions, but reduced in size from no task to familiar, and from familiar to unfamiliar concurrent finger movement.

## Experiment 5: familiar and unfamiliar arm movement

This final experiment applied familiar and unfamiliar arm movement to the encoding phase of the remembering instructions task. We again explored whether concurrent movement, and particularly when this was unfamiliar, would impact on working memory, and more so for enacted recall.

### Method

#### Participants

Overall, 24 right-handed adults (aged 18–26 years, *M* = 21.04 years, *SD* = 1.33; 22 females and 2 males).

The primary task materials were the same as those used in the previous experiments.

#### Materials, design, and procedure

As in Experiment 4, this experiment used a 2 (recall mode: verbal, action) × 3 (concurrent task: no task, familiar task; tracing the letter “A”), complex task (tracing an inverted A, i.e., “∀”), repeated measures design. Experimental and trial structures were implemented as in the previous experiments. Trial procedure was also identical, with the exception that Experiment 5 required tracing movements with the right arm.

### Results

**Figure 5.**
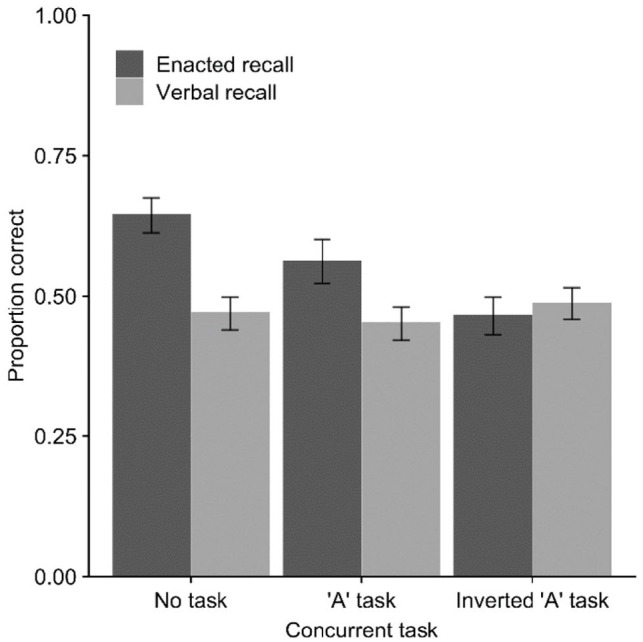
Mean proportion of action–object pairs correct (with *SE*) in Experiment 5 across verbal and enacted recall modes and concurrent movement task conditions.

Mean proportion of action–object pairs recalled in the correct serial position is displayed in Figure 5.

The overall 3 × 2 ANOVA indicated a significant effect of recall mode, *F*(1, 23) = 21.86, *p* < .001, $\eta_{p}^{2}=.49$ , *BF*_10_ > 10,000, with superior performance under enacted (*M* = 0.56, *SE* = 0.03) relative to verbal (*M* = 0.47, *SE* = 0.03) recall conditions. The main effect of concurrent task was significant, Greenhouse–Geisser corrected, *F*(1.61, 37) = 11.35, *p* < .001, $\eta_{p}^{2}=.33$ , *BF*_10_ = 110.76, with further comparisons revealing that recall in the no-task condition (*M* = 0.56, *SE* = 0.03) was higher than in both the familiar, (*M* = 0.51, *SE* = 0.03), *t*(23) = 2.92, *p* = .011, *d* = 0.60, *BF*_10_ = 6.38, and unfamiliar, (*M* = 0.48, *SE* = 0.03), *t*(23) = 4.72, *p* < .001, *d* = 0.96, *BF*_10_ = 74.76, movement conditions, which did not themselves differ, *t*(23) = 1.80, *p* = .08, *d* = 0.37, *BF*_10_ = 1.02. The interaction between recall mode and concurrent task was also significant, *F*(2, 56) = 34.94, *p* < .001, $\eta_{p}^{2}=.60$ , *BF*_10_ > 10,000.

For the 2 × 2 ANOVA comparing no task with familiar (“A”) movement, there was an effect of recall mode, *F*(1, 23) = 43.40, *p* < .001, $\eta_{p}^{2}=.65$ , *BF*_10_ > 10,000, concurrent task, *F*(1, 23) = 5.94, *p* = .023, $\eta_{p}^{2}=.21$ , *BF*_10_ = 4.23, and the interaction, *F*(1, 23) = 7.58, *p* = .011, $\eta_{p}^{2}=.25$ , *BF*_10_ = 0.96, though this latter finding was not supported by Bayesian analysis (*BF*_01_ = 1.04). For the comparison of no task with unfamiliar (inverted “A” movement), there was an effect of recall mode, *F*(1, 23) = 18.75, *p* < .001, $\eta_{p}^{2}=.45$ , *BF*_10_ = 90.70, and concurrent task, *F*(1, 23) = 22.75, *p* < .001, $\eta_{p}^{2}=.50$ , *BF*_10_ = 169.23, and a significant interaction, *F*(1, 23) = 51.82, *p* < .001, $\eta_{p}^{2}=.69$ , *BF*_10_ > 10,000.

This is reflected in the comparison of recall modes which indicated an advantage for enacted versus verbal recall in the no-task condition, *M* = 0.64 versus *M* = 0.47, *t*(23) = 7.28, *p* < .001, *d* = 1.49, *BF*_10_ > 10,000, and in the familiar task condition, *M* = 0.56 versus *M* = 0.45, *t*(23) = 4.38, *p* < .001, *d* = 0.89, *BF*_10_ = 133.98, but not in the unfamiliar task condition, *M* = 0.47 versus *M* = 0.49, *t*(23) = −1.07, *p* = .30, *d* = 0.22, *BF*_10_ = 0.36.

### Discussion

Experiment 5 examined whether familiarity of concurrent arm movement would impact on memory for instructions, and in particular the enacted recall advantage. As with Experiment 4, movement pattern familiarity did indeed impact on performance, with concurrent unfamiliar movement causing relatively larger disruptive impacts on recall accuracy. Furthermore, this effect varied as a function of recall mode, with the effect of an unfamiliar action being substantially greater for enacted responses. As such, this reinforces the conclusion from previous experiments for a specific motor interference effect rather than an interpretation purely in terms of a general dual-task executive cost. In particular, the enacted recall advantage was not observed when participants performed an unfamiliar movement pattern during encoding of the instruction sequences.

## Combined analysis of Experiments 4 and 5

The 2 × 3 × 2 mixed ANOVA indicated a significant effect of recall mode, *F*(1, 46) = 78.64, *p* < .001, $\eta_{p}^{2}=.63$ , *BF*_10_ > 10,000, with better performance for enacted (*M* = 0.57, *SE* = 0.02) than verbal (*M* = 0.46, *SE* = 0.02) recall. The main effect of concurrent task was also significant after Greenhouse–Geisser correction, *F*(1.74, 80.03) = 34.19, *p* < .001, $\eta_{p}^{2}=.43$ , *BF*_10_ > 10,000, with pairwise comparisons showing better recall in the no-task condition (*M* = 0.55, *SE* = 0.02) than in either the familiar, (*M* = 0.51, *SE* = 0.02), *t*(47) = 3.75, *p* < .001, *d* = 0.54, *BF*_10_ = 247.48, or the unfamiliar, (*M* = 0.47, *SE* = 0.02), *t*(47) = 8.17, *p* < .001, *d* = 1.18, *BF*_10_ > 10,000, conditions, which also differed from each other, *t*(47) = 4.94, *p* < .001, *d* = 0.71, *BF*_10_ = 982.69, with the unfamiliar task causing most disruption. The interaction between recall mode and concurrent task was also significant, *F*(2, 92) = 45.57, *p* < .001, $\eta_{p}^{2}=.50$ , *BF*_10_ > 10,000, with the concurrent tasks disrupting recall of enacted more than spoken responses. There was neither main effect of experiment nor any two-way interactions with other factors (*F* < 3.1, *p* > .085, $\eta_{p}^{2}<.065$ , *BF*_10_ < 0.3). However, there was a significant three-way interaction between recall mode, concurrent task, and experiment, *F*(2, 92) = 5.12, *p* = .008, $\eta_{p}^{2}=.10$ , *BF*_10_ = 1.12, though with only weak BF support.

For the 2 × 2 × 2 ANOVA comparing no task with familiar movement, there was an effect of recall mode, *F*(1, 46) = 118.80, *p* < .001, $\eta_{p}^{2}=.72$ , *BF*_10_ > 10,000, and concurrent task, *F*(1, 46) = 13.79, *p* < .001, $\eta_{p}^{2}=.23$ , *BF*_10_ = 107.02, and the recall by task interaction, *F*(1, 46) = 20.19, *p* < .001, $\eta_{p}^{2}=.32$ , *BF*_10_ = 15.52. There was no main effect of movement amplitude (i.e., finger vs arm) or interaction with any other factor (*F* < 1, *p* > .5, $\eta_{p}^{2}<.01$ , *BF*_10_ < 0.5).

For the comparison of no task with unfamiliar movement, there was an effect of recall mode, *F*(1, 46) = 70.40, *p* < .001, $\eta_{p}^{2}=.61$ , *BF*_10_ > 10,000, and concurrent task, *F*(1, 46) = 65.83, *p* < .001, $\eta_{p}^{2}=.59$ , *BF*_10_ > 10,000, and a significant interaction, *F*(1, 46) = 74.47, *p* < .001, $\eta_{p}^{2}=.62$ , *BF*_10_ > 10,000. There was no main effect of movement amplitude or interaction with task, (*F* < 1, *p* > .5, $\eta_{p}^{2}<.01$ , *BF*_10_ < .5), but we did observe a significant two-way interaction between recall mode and movement amplitude, *F*(1, 46) = 5.91, *p* = .019, $\eta_{p}^{2}=.11$ , *BF*_10_ = 6.75. There was also a significant three-way interaction, *F*(1, 46) = 5.03, *p* = .030, $\eta_{p}^{2}=.10$ , *BF*_10_ = .974, though this latter finding was not supported by the BF (*BF*_01_ = 1.03).

Finally, comparison of recall modes indicated an advantage for enacted versus verbal recall advantage for the no-task condition, *M* = 0.65 versus *M* = 0.46, *t*(47) = 11.26, *p* < .001, *d* = 1.63, *BF*_10_ > 10,000, and the familiar task condition, *M* = 0.57 versus *M* = 0.45, *t*(47) = 7.50, *p* < .001, *d* = 1.08, *BF*_10_ > 10,000, but not in the unfamiliar task condition, *M* = 0.48 versus *M* = 0.45, *t*(47) = 1.81, *p* = .077, *d* = 0.26, *BF*_10_ = 0.70.

To summarise, the combined analysis of Experiments 4 and 5 provides clear evidence for a recall mode by concurrent task interaction when comparing the no-task condition with either familiar or unfamiliar movement, but this is stronger for the latter concurrent task condition. Thus, confirming the outcomes from the separate experiments, the enacted recall advantage was reduced by concurrent movement, particularly when this was unfamiliar. There is also some evidence for an interaction with movement scale when performing an inverted “A” movement, whereby the experiment involving concurrent arm movement (Experiment 5) resulted in a larger decline in the enacted recall effect, but this three-way interaction was not supported by Bayesian analysis.

## General discussion

We set out to use dual-task methodology to explore how working memory supports the planning of forthcoming actions. To achieve this, we measured the interfering effect of different motoric secondary tasks while listening to instructions to perform a series of actions on a set of objects, comparing the accuracy of physical enactment with that of verbal recall. Given the auditory-verbal nature of instruction presentation, we assume that baseline performance in this paradigm is set by the verbal component of working memory, with motor representations providing supplementary support that enhances performance and enables action planning. We were particularly interested in the effects of various secondary motor tasks on the resultant “enaction advantage”; the observation that enacting instructions is more accurate than recalling them (e.g., Gathercole et al., 2008). Prior to the present investigation, direct evidence about the resources specialised for planning forthcoming actions consisted principally of results showing the enaction advantage can be reduced by a secondary task that involves making movements (Jaroslawska et al., 2018), but not by tasks loading verbal, executive, or visuospatial components of working memory (Yang et al., 2014, 2016). More specifically, Jaroslawska et al. (2018) found that a secondary task requiring gross, body-level movements removed the enaction advantage, whereas one involving configural movements of the hand did not. From this, they concluded that the motor component of working memory is primarily concerned with body-level movements. However, the observed difference was small, based on separate experiments, and potentially confounded with other factors, such as movement complexity. The present experimental series therefore started by comparing the interfering effects of fine (finger) and gross (arm) motor tasks with movement complexity controlled. We then went on to explore the effects of varying the complexity and familiarity of the secondary movement task, with the idea that the sensitivity of the enaction advantage to the manipulations of scale, complexity, and familiarity would reflect the characteristics of the motoric component of working memory.

At the broadest level, our results are straightforward in that all five experiments replicated the enaction advantage and confirmed that it is reduced when a secondary motor task is performed during the instruction phase. This greatly extends the limited previous evidence for ascribing the enaction advantage to a limited capacity motoric component of working memory. We conclude that planning to perform a series of actions while listening to the instructions draws on the same pool of resources as carrying out a concurrent motoric task. We also found that manipulations of concurrent movement complexity (Experiments 2 and 3) and familiarity (Experiments 4 and 5) influenced the performance and reduced the enaction advantage. Representations of movements comprising more elements will presumably be more complex than representations of movements with fewer elements and will take up more capacity within the motoric component, leaving less available for other ongoing activities, such as enhancing retention of actions awaiting performance. Similarly, representations of unfamiliar actions will be more complex than representations of familiar actions, given that familiar actions are likely to benefit from chunking through extended practice (Lashley, 1951; Logan & Crump, 2011).

However, this study provided only limited evidence to support Jaroslawska et al. (2018) that any motor contribution specifically reflects gross movement. Experiment 1 broadly replicated the findings from this earlier study, with an interaction between recall mode and concurrent task for arm but not finger movement. This was not supported by Bayesian analysis though, and the enacted recall advantage remained intact (albeit reduced in size). Experiments 2 and 3 showed the enactment advantage was sensitive to the complexity of concurrent movements but this was independent of and unaffected by their scale. Similarly, for Experiments 4 and 5, movement scale had no impact when using a familiar movement. However, the enaction advantage was abolished by arm (Experiment 5) and not finger (Experiment 4) concurrent unfamiliar movement. This latter finding represents a replication of those reported by Jaroslawska et al. (2018), though the Bayesian support for the interaction in this case was again weak. Based on these findings, we might conclude that the requirement for concurrent movement, that is, both gross and unfamiliar is important in causing the inability to set up a motor representation and therefore abolishing the enaction advantage. Alternatively, the outcomes from Experiment 5 might reflect an inability to detect a motor component that remains but is masked by the more efficient verbal component when concurrent demands increase. In this context, we note in retrospect that the suggestion from Jaroslawska et al. (2018) that the motor resources of working memory are primarily concerned with body-level rather than fine-grain movements are somewhat oversimplistic. In practice, movements often involve a combination of gross and fine scales (as in this study) which would introduce a further problem in coordinating the two systems. While the gross–fine distinction is not straightforward, our data do not rule it out; they do not, however, provide strong support for an emphasis of gross over fine motor representation in working memory.

Overall, and especially when we consider the combined analyses of Experiments 2 and 3, and Experiments 4 and 5, the current study provides strong evidence that certain types of concurrent movement task can reduce and even remove the otherwise consistent enacted recall advantage. This would indicate that planned and current actions compete for the resources of a motoric component in working memory. How might theoretical approaches explain our findings? At the broad level, Laird et al. (2017) suggest what they term a “standard model” of the mind based on the SOAR architecture (Laird et al., 1986; Newell, 1992). This includes perceptual and motor buffers within working memory that are accessed and modified by distinct perceptual and motor modules. Within working memory frameworks that incorporate multiple subcomponents, a recent iteration of the time-based resource-sharing (TBRS) approach sets out an architecture that includes phonological and visuospatial input buffers, an episodic buffer for holding the core working memory representation, and separate motor output buffers for action and speech (Barrouillet & Camos, 2014, 2021). The multicomponent system described by Logie et al. (2021) does not label specific subcomponents per se but describes how task performance is supported by a form of “cognitive toolbox,” in which information drawn from sensory input and activated prior knowledge is retained in a range of domain-specific stores that can each interact and contribute to working memory “capacity.”

Our own current iteration of a multicomponent model (Baddeley et al., 2021) emphasises the flow of information into working memory together with its executive control. At this point, it is important to outline the way in which the phonological and visuospatial subsystems are currently conceived. From the initial concept of simple temporary phonological or visuospatial storage systems, the two are now assumed to operate in a more complex way, located at the confluence of streams of visuospatial and acoustic-phonological information. Each can combine and compress the information from multiple streams into broad visuospatial or phonological representations, which may then be combined with each other and data from LTM into a multidimensional form and made available through the episodic buffer. In short, these representations within the episodic buffer combine visuospatial, phonological, and potentially semantic information from long-term memory and in a form that is available to conscious awareness.

The concept of a phonological loop includes separate auditory and articulatory processes, capable of storing perceptual information (the “inner ear”) and motor information (the “inner voice”), respectively (Baddeley & Lewis, 1981; Mattys & Baddeley, 2019; Mattys et al., 2018; Norris et al., 2018; Vallar & Papagno, 2002). In an analogous way, the current evidence may be incorporated as part of a more detailed specification within the visuospatial sketchpad. This might involve a visuospatial input store linked to a motor output store concerned with developing and holding plans for immediate future action. Such a view could be regarded as a development of Logie’s (1995) concept of an Inner Scribe (see also Logie et al., 2001). It would provide a locus for the enacted recall benefit observed in this and previous studies, for the benefits of self-enactment and demonstration during encoding (e.g., Allen et al., 2020; Allen & Waterman, 2015; Coats et al., 2021; Waterman et al., 2017; Yang et al., 2015, 2017) and the recent observation of children’s enhanced recall following the explicit instruction to imagine performing each action during encoding (Yang et al., 2021).

One way of thinking about a motoric working memory component is as a two-stage process broadly analogous to the way the phonological loop has been described as operating in immediate serial recall. In that task, verbal responses are assumed to be simultaneously active in the plan for recall and during their sequential output, a process that can be explained in terms of the repeated applications of a competitive queueing mechanism (Hurlstone et al., 2014). However, even if planning a series of actions is analogous to serial verbal recall, translating verbal instructions into a plan for a series of actions on physical objects in different spatial locations must be considerably more complex and multidimensional than merely repeating a verbal sequence. The current study therefore highlights a major gap in the multicomponent model that concerned with action control. Although it is seen as providing an interface between cognition and action (Baddeley, 2007, 2012; Baddeley et al., 2021), our approach has so far been dominated by the interface between perception and executive control. This may have resulted from a tendency to focus on studies using verbal material, such as digits, words, and text, for which it makes little difference to retention whether responses are made verbally or manually by key pressing or cursive writing. The attempt to understand the processes involved in following instructions to perform specific actions forces us to go beyond verbalised responding with results that are important in suggesting the need to extend the current framework. This is not an issue that solely applies to the multicomponent model, of course. It will be of important for the working memory field in general to explore this issue, from both methodological and theoretical perspectives. Such developments would be in line with the suggestion that motor control should be considered when examining cognition and behaviour (Rosenbaum, 2005; Rosenbaum & Feghhi, 2019), and the growing literature on the interaction between motor learning and working memory (e.g., Raw et al., 2019) and the involvement of systems concerned with motor control in working memory (see Tomasino & Gremese, 2016 for a review). This evidence suggests that a mapping of action-related areas of the primary motor cortex onto a range of other aspects of working memory may in future prove fruitful.

An alternative solution is that proposed by Jones, Macken, and colleagues (e.g., Hughes & Jones, 2005; Jones et al., 2006; Jones & Macken, 2004, 2018; Macken et al., 2015) who treat working memory as a direct mapping of perceptual organisation onto output planning and reject the need to assume buffers holding abstract, post-categorical representations. Short-term memory phenomena are viewed as properties of an object-oriented action system in which the opportunistic co-opting of perceptual-motor processes enables output plans to “pick up” residual information directly from the input stream (Jones et al., 2006, p. 278). This approach has been explored in detail in the context of the speech motor system, and Jones and Macken (2018) note that it could apply to effector systems responsible for hand and arm movements too. At first sight, it fails to explain the action advantage and its reduction by concurrent motor movement. This is because in sensorimotor terms, the link between hearing and speaking should allow for a more direct “pick up” from perception to output as compared to that between hearing and action (McLeod & Posner, 1984). According to Jones and Macken (2018), “the effector system that usually can most readily be co-opted for the apprehension of verbal material is the speech motor system” (p. 352). Thus, the object-oriented action system approach would seem to predict an advantage for verbal recall over enactment following spoken presentation, the exact opposite of what we find. However, we acknowledge that this analysis is simplistic and ignores subtleties of the perceptual-motor account (see e.g., Macken et al., 2016) that could be invoked to explain our findings. We agree that exploring to what extent a purely perceptual-motor account can capture critical findings in the short-term and working memory literature is a useful exercise that is certainly relevant to the question of how instruction sequences are encoded, retained, and implemented. Finally, the suggestion that perceptual and motor skills are “co-opted” to support short-term and working memory performance is not a controversial one and is in fact broadly accepted by multicomponent frameworks.

Our broad interpretation of the present findings is that motoric information can be incorporated into working memory to support planned enactment of verbal instruction, and that this process can be disrupted by concurrent movement. While we have speculated on how such findings might be captured by existing theoretical approaches, there of course remain many details that are yet to be established. This includes the question of whether the enactment advantage arises “within” working memory itself, or whether this system co-opts and stores outputs derived from motor planning processes that operate externally to working memory. Along similar lines, further work might explore whether working memory for instructions and the enactment effect are sensitive to variations along dimensions, such as movement scale, familiarity, and complexity disrupt, due to interference with the initial creation or subsequent storage of enactment plans. One possibility is that motor planning interfaces with working memory in a way that is analogous to how simple visual feature binding may initially emerge automatically through perceptual processes before being held in a consciously accessible form in working memory (e.g., Baddeley et al., 2011; Hitch et al., 2020). Indeed, in addition to enriching the mnemonic representation through development of a motor plan, preparing for intended movement might also aid encoding and storage by binding information of different types into a coherent, global, gesture, or representation (e.g., Yang et al., 2016).

In conclusion, we have attempted to use dual-task methodology to explore the practically important topic of how we respond to spoken instructions and how speech may be translated into actions. Specifically, we examined the proposal that this involves some form of temporary representation of future actions that is separate from their spatial or verbal form. Over five experiments, we find evidence for the assumption of temporary motoric storage in a system whose capacity is limited by both the complexity and familiarity of the concurrent activity. We suggest that this highlights the need for a better understanding of the link between working memory and action. Broad models of action control that bring together research on perception, motor control with evidence from neuropsychology have already been proposed (e.g., Frith et al., 2000) and have been linked to the issue of working memory and the control of action (See Baddeley, 2007, Chapter 17). There is, however, a considerable gap between such models and our current models of working memory. We regard the present studies as a step towards beginning to bridge that gap. We suggest that any attempt to close this gap should adopt a broad framework combined with a series of steps that investigate the way in which the various components of working memory, peripheral, and central combine to achieve its various functions and ensure continuity and coherence between recent and upcoming actions and events.

## Supplemental Material

sj-docx-1-qjp-10.1177_17470218221079848 – Supplemental material for Translating words into actions in working memory: The role of spatial-motoric coding Click here for additional data file. Supplemental material, sj-docx-1-qjp-10.1177_17470218221079848 for Translating words into actions in working memory: The role of spatial-motoric coding by Guangzheng Li, Richard J Allen, Graham J Hitch and Alan D Baddeley in Quarterly Journal of Experimental Psychology
