# Supplementary material for: Translating words into actions in working memory: The role of spatial-motoric coding
Source: Q J Exp Psychol (Hove). 2022 Mar 3;75(10):1959–75. doi: 10.1177/17470218221079848 (PMC9424718; doi:10.1177/17470218221079848)
Supplement: sj-docx-1-qjp-10.1177_17470218221079848 – Supplemental material for Translating words into actions in working memory: The role of spatial-motoric coding [file sj-docx-1-qjp-10.1177_17470218221079848.docx]

Supplementary materials for:

Translating words into actions in working memory: the role of spatial-motoric coding

Guangzheng Li, Richard J. Allen, Graham J Hitch, & Alan D. Baddeley

The below data show proportion correct recall of actions and objects (calculated separately) for each experiment.


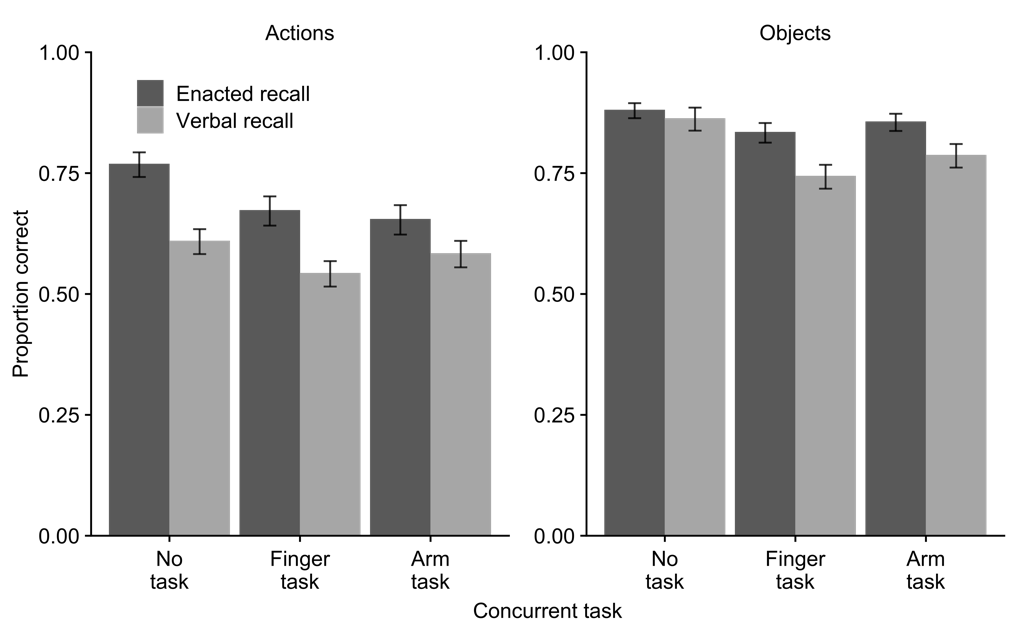
Experiment 1

Figure S1. Mean proportion of actions and objects correct (with SE) in Experiment 1 across verbal and enacted recall modes and concurrent movement task conditions


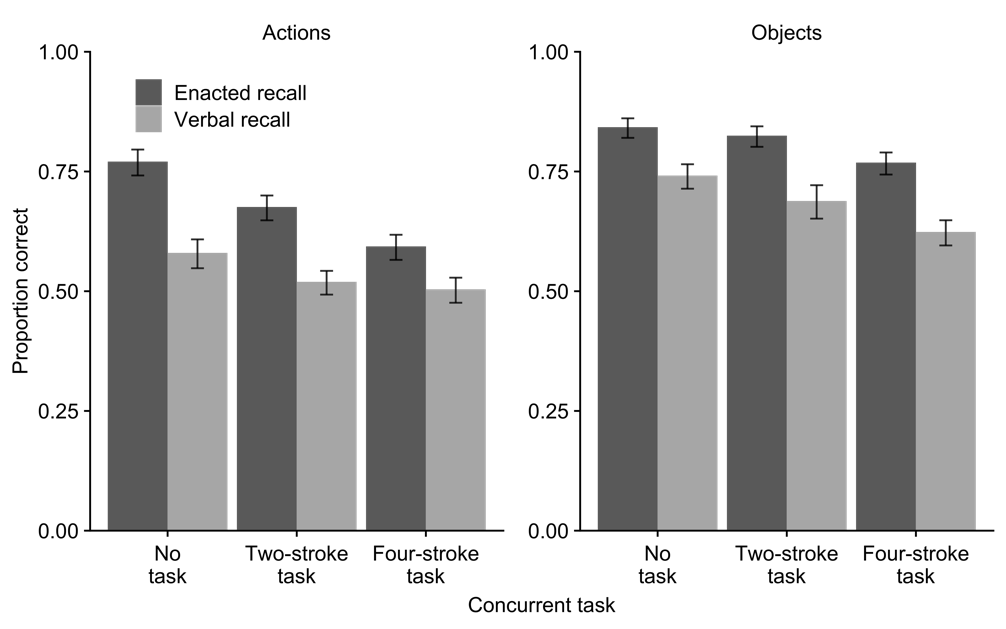
Experiment 2

Figure S2. Mean proportion of actions and objects correct (with SE) in Experiment 2 across verbal and enacted recall modes and concurrent movement task conditions


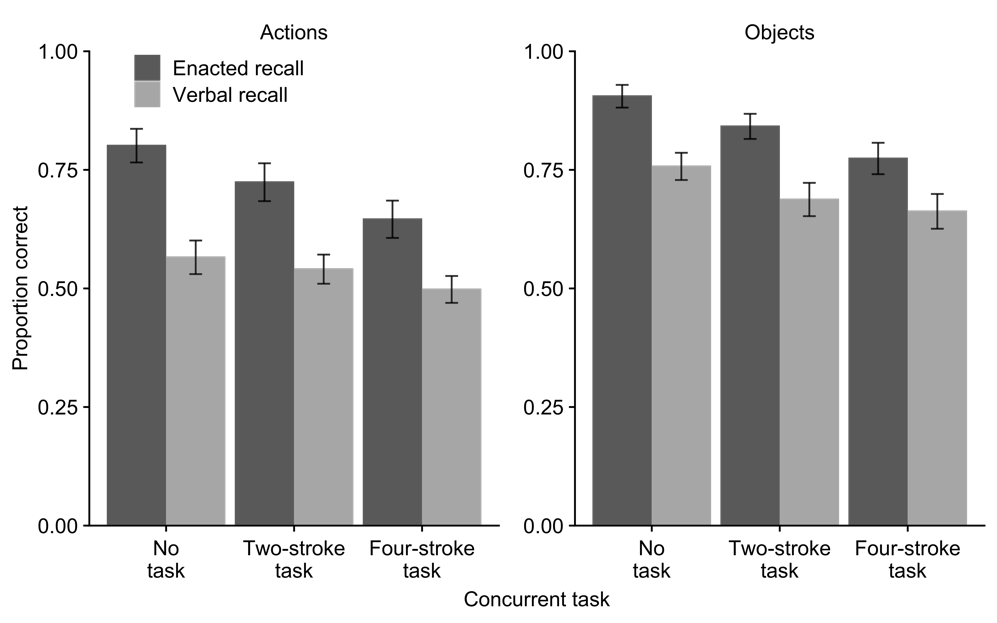
Experiment 3

Figure S3. Mean proportion of actions and objects correct (with SE) in Experiment 3 across verbal and enacted recall modes and concurrent movement task conditions


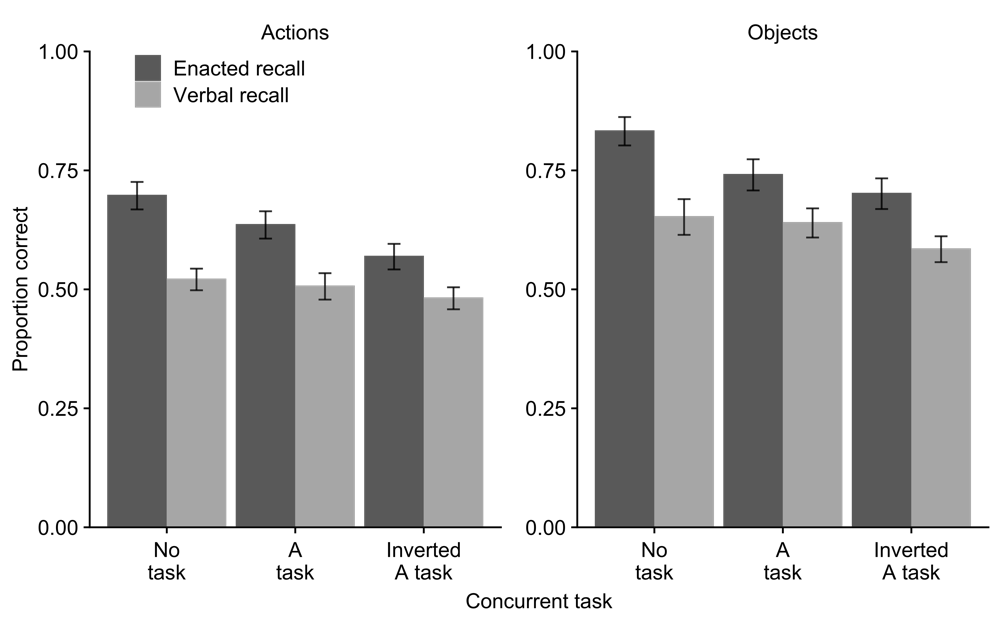
Experiment 4

Figure S4. Mean proportion of actions and objects correct (with SE) in Experiment 4 across verbal and enacted recall modes and concurrent movement task conditions


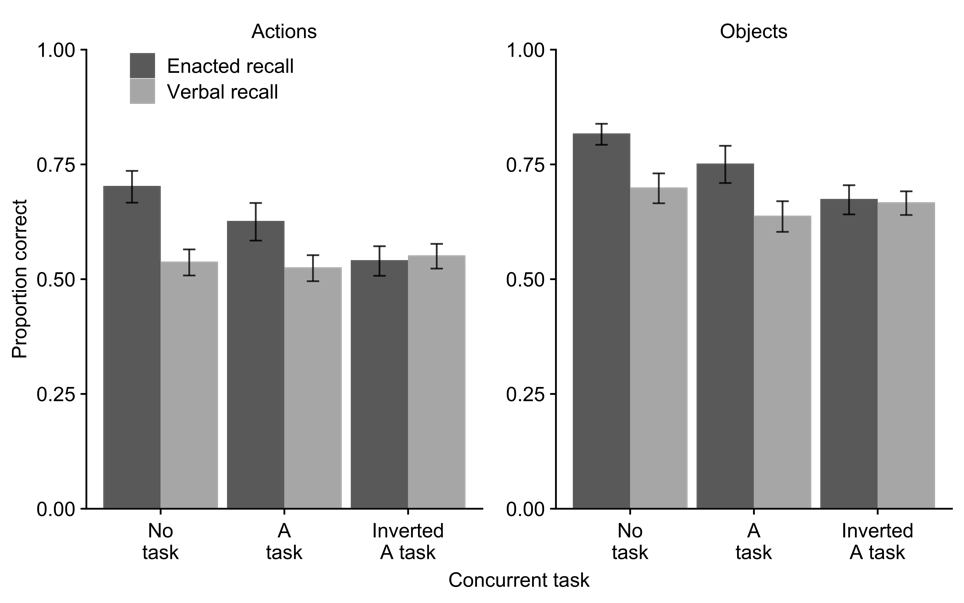
Experiment 5

Figure S5. Mean proportion of actions and objects correct (with SE) in Experiment 5 across verbal and enacted recall modes and concurrent movement task conditions

Analysis summary

A series of 3x2x2 (concurrent task x recall mode x feature type) Bayesian ANOVA were carried out, with the aim of examining whether the two main factors of interest were affected by type of feature type (i.e., action vs. object).

In all five experiments, the main effect of feature type was supported (BF_10_ >10,000 in each case), which recall being more accurate for objects compared to actions. This consistent and strongly supported outcome replicates previous findings (Allen et al., 2020).

Regarding the presence or absence of interactions, in Experiment 1 there is evidence that feature type interacts with recall mode (BF_10_ =8.53), and for a three-way interaction (BF_10_ = 3.23). This seems to be driven mainly by the absence of an enacted recall effect for objects under no-task conditions (BF_10_ = .282), a finding that was not replicated in the subsequent experiments (BF_10_ >100 for this comparison in Experiments 2-5). For the remaining experiments, there is no informative supporting evidence for two- or three-interactions between feature type and any other factor (BF_10_ <3 for all experiments).

Thus, overall, there is no clear indication from this separate analysis of actions and objects that concurrent task and recall mode interact differentially with type of feature from the instruction sequences.
